# Genetic diversity of *Hepatozoon* (Apicomplexa) from domestic cats in South Africa, with a global reassessment of *Hepatozoon felis* diversity

**DOI:** 10.4102/jsava.v90i0.1747

**Published:** 2019-06-20

**Authors:** David J. Harris, Ali Halajian, Joana Santos, Kgethedi M. Rampedi, Raquel Xavier

**Affiliations:** 1 CIBIO/InBIO, Centro de Investigação em Biodiversidade e Recursos Genéticos, Vairão, Portugal; 2 Departamento de Biologia, Faculdade de Ciências da Universidade do Porto, Porto, Portugal; 3 Department of Biodiversity, University of Limpopo, Sovenga, South Africa; 4 Agricultural Research Council Animal Production Institute, Irene, South Africa

**Keywords:** 18S rRNA, hepatozoonosis, *Felis catus*, *Sarcocystis*, phylogeny

## Abstract

Genetic diversity within partial 18S rRNA sequences from *Hepatozoon* protozoan parasites from domestic cats in South Africa was assessed and compared against published data to assess global biogeographic patterns. Multiple distinct haplotypes of *Hepatozoon felis* were identified, as well as an unrelated *Hepatozoon* lineage. *Hepatozoon felis* genetic diversity globally is very high, indicating a likely complex of species. The recently described *Hepatozoon apri* from wild boars is closely related to some lineages of *H. felis. Sarcocystis* and *Babesia* parasites were also detected. Since *Hepatozoon felis* is apparently a species complex, potential differences between genetically distinct forms need to be assessed. The finding of an unrelated *Hepatozoon* indicates that felids can be infected by more species of *Hepatozoon* than currently known, and that trophic interactions may increase the number of *Hepatozoon* species found in carnivores. Genetic screening again is demonstrated to identify previously unrecognised parasites from vertebrate hosts.

## Introduction

Species of the genus *Hepatozoon* are apicomplexan parasites infecting most terrestrial vertebrate groups, having haematophagous arthropods as the definitive hosts. Recent phylogenetic assessments have shown a complex pattern of relationships, with the genus *Karyolysus* forming a clade within a paraphyletic *Hepatozoon*, although parasites from most mammalian carnivores form a well-defined genetic lineage (Maia et al. 2016). Clinical features of hepatozoonosis are widely reported in canids, but the situation in felids is less clear. *Hepatozoon* parasites have been identified in many different felids, either through identification of gamonts in blood smears or using DNA sequencing techniques. Reported hosts include lynx, cheetahs, lions, jaguar, tigers, wildcats and domestic cats (Baneth et al. 1998a). *Hepatozoon* parasites were first reported in a domestic cat from India over one hundred years ago, and until recently, only *Hepatozoon felis* was considered to occur in felids, with the exception of rare records of *Hepatozoon canis*, a common parasite of canids (Williams et al. 2014). *Hepatozoon felis* was recently recovered from ticks infesting humans in Turkey (Karasartova et al. 2018). Reported infection rates vary, for example, from only 0.57% of cats infected from a sample of 1229 from Israel (Baneth et al. 1998b) to 37.9% of 174 cats from Cyprus (Attipa et al. 2017). Several authors have noted ‘*H. felis*-like’ infections, with parasites showing genetic similarity to *H. felis* but some level of morphological or genetic distinction (Giannitti et al. 2012). Recently, a new species was described from the European wildcat, *Hepatozoon silvestris* (Hodžić et al. 2017:650–661), which was then also found in domestic cats in Italy (Giannelli et al. 2017). However, it is not clear how the different genetic lineages of *Hepatozoon* from felids are related, while sampling from many geographic regions is limited. This is essential information, because, in canids, different *Hepatozoon* species can have very different impacts on the health of the host and thus have a different veterinary importance (Vincent-Johnson et al. 1997). Similarly, a recent study identified subclinical infections with the haemoparasite *Cytauxzoon felis* in cats, suggesting the existence of different strains that may vary in pathogenicity, although this link was not identified with the ITS1 genetic loci (Pollard et al. 2017). Whether different genetic forms of *Hepatozoon* in felids have different pathological effects remains unknown, although a fatal infection with *H. silvestris* has recently been reported in a domestic cat (Kegler et al. 2018). Furthermore, ‘*H. felis*-like’ genetic lineages were recently identified in a genet, *Genetta genetta* (Harris et al. 2017), and a white-tailed mongoose, *Ichneumia albicauda* (Harris et al. 2018), both from South Africa, increasing the number of mammalian families known to be potentially infected. Adding to the already complex taxonomy, *Hepatozoon apri* was recently described from wild boars in Japan, with haplotypes appearing to be more closely related to *H. felis* than *H. silvestris* (Yamamoto et al. 2017). Similarly, *Hepatozoon martis* was recently described from martens, closely related to *H. apri* (Hodžić et al. 2018). Other recent studies have also shown that *H. felis* and *H. silvestris* may not be closely related (De Azevedo Gomes et al. 2017). Additionally, one study focusing on *Hepatozoon* from canids and rodents that included three haplotypes from *H. felis* showed that the species as currently constituted is not monophyletic (Maia et al. 2014).

Despite the recently identified distinct lineages and species, no studies have yet tried to determine the phylogenetic relations between all known *H. felis* haplotypes and those of closely related species. The aims of this study were to gather the first genetic data on *Hepatozoon* from domestic cats from South Africa and to decipher global patterns of diversity, by assessing the genetic variation within *H. felis* and *H. felis*-like parasites.

## Research methods and design

Sampling consisted of specimens either collected as road kills or from tissue banks from various collections in South Africa. Sixteen individuals were assessed, with muscle and/or liver tissues being tested (Table 1). The molecular approach followed standard procedures used in other screening studies, which have already shown that muscle tissue can be effectively used to extract DNA from apicomplexan parasites (Harris et al. 2012). DNA was extracted from different tissues using high salt procedures, which is as effective as commercial kits for extracting parasite DNA (Maia et al. 2014). Polymerase chain reaction (PCR) amplification of part of the 18S rRNA gene was performed using the primers HepF300 and HepR900 (Ujvari et al. 2004) with 35 cycles consisting of 95 ºC (45 seconds [s]), 60 ºC (45 s) and 72 ºC (90 s). Negative and positive controls were run with each reaction, and the products were sequenced by a commercial company (Genewiz, United Kingdom). Electropherograms were checked by eye, and compared against published sequences on GenBank using BLAST. All new sequences were submitted to GenBank (MK301457 to MK301462).

**TABLE 1:** Samples screened in this study.

Code	Tissue	Locality within South Africa
CatR71014	**M**/L	Mankweng, Limpopo Province
CatULN14	M/**L**†	University of Limpopo, Limpopo Province
CatUL715	M/L	University of Limpopo, Limpopo Province
Cat17915	M/**L**†	University of Limpopo, Limpopo Province
CatRK66	M	Road N3
Cat9152	**M**†/**L**†	Mankweng, Limpopo Province
CatUL115	M/**L**	University of Limpopo, Limpopo Province
Cat8416	**M**†	Fetakgomo, Limpopo Province
Cat10162	**M**/**L**	University of Limpopo, Limpopo Province
Cat1016	**M**†/L	University of Limpopo, Limpopo Province
Cat1116	M/**L**†	Mankweng, Limpopo Province
Cat16	M/**L**	University of Limpopo, Limpopo Province
CatUL1216	M/L	University of Limpopo, Limpopo Province
Cat12162	M/L	University of Limpopo, Limpopo Province
CatUL816	**M**/**L**	University of Limpopo, Limpopo Province
Cat6717	M	Mogalakwena, Limpopo Province

Note: Bold, positive samples.

M, muscle tissue; L, liver tissue.

† , Positive *Hepatozoon felis* sequences.

New sequences were aligned against published data from GenBank using the ClustalW software implemented in Geneious 4.8.5 (Biomatters Ltd). Sequences used are given in Supplementary Table 1. Phylogenetic relationships were estimated using Bayesian inference (BI) and maximum likelihood (ML). Models of sequence evolution (GTR+G, 1 partition) were selected by PartitionFinder 1.1.1 (Lanfear et al. 2012:1695–1701). Maximum likelihood estimates were performed using RaxML (Stamatakis 2014) with ten random replicates and supported nodes identified through 1000 nonparametric bootstrap iterations. Bayesian inference was implemented using MrBayes version 3.2.6 (Huelsenbeck & Ronquist 2001) and run for one million generations. After 25% burn-in, remaining trees were combined in a 50% majority rule consensus. *Karyolysus paradoxa* was used to root the tree. When few characters are available because of the slow evolving nature of the genes examined, networks can also be appropriate ways to represent genetic variation (Posada & Crandall 2001). We therefore estimated a phylogenetic network using the statistical parsimony approach implemented in TCS (Clement et al. 2000), both with a 95% probability criterion for connections and with a 20 connection step limit to visualise relationships of more divergent haplotypes. Although longer fragments were generated in this study, as many of the fragments available on GenBank were much shorter, a final alignment of 255 bps was used.

## Results

**FIGURE 1:**
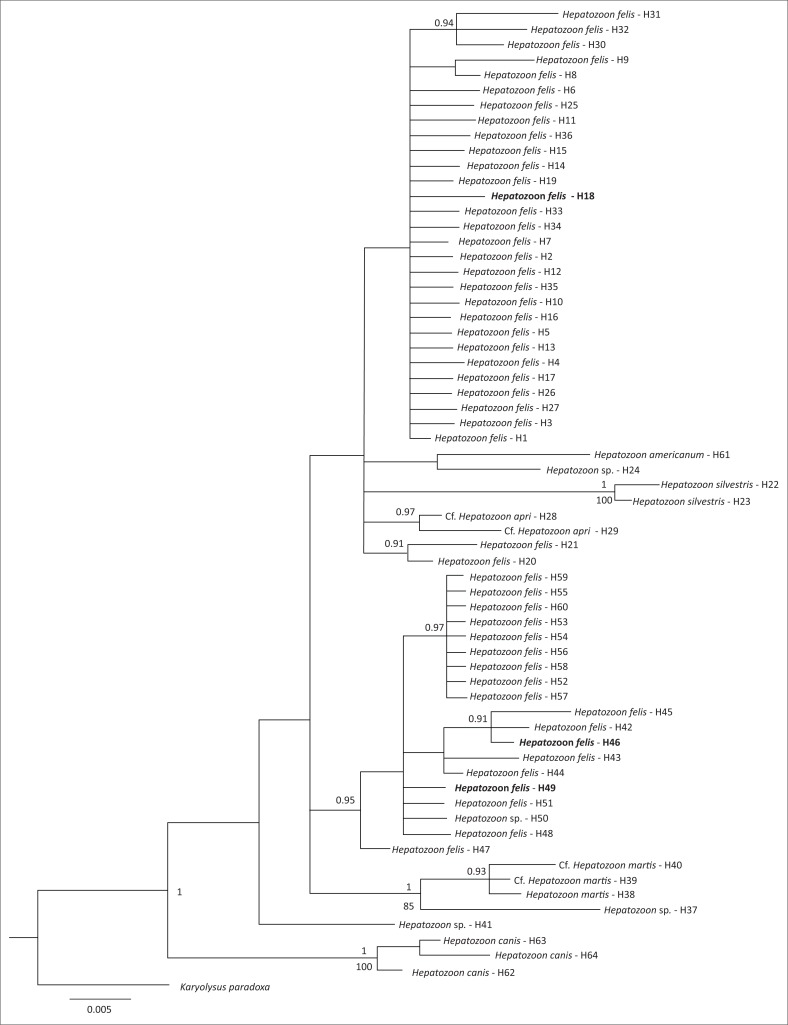
Estimate of relationships derived from a Bayesian inference analysis. Sequences derived for this study are in bold. Codes (H1-H64) refer to Supplementary material for GenBank numbers and additional details. The same code numbers are used in Figure 2. Posterior probabilities (> 0.9) and ML bootstrap support (> 85%) are indicated above and below nodes, respectively. The tree was rooted using *Karyolysus paradoxa*.

**FIGURE 2:**
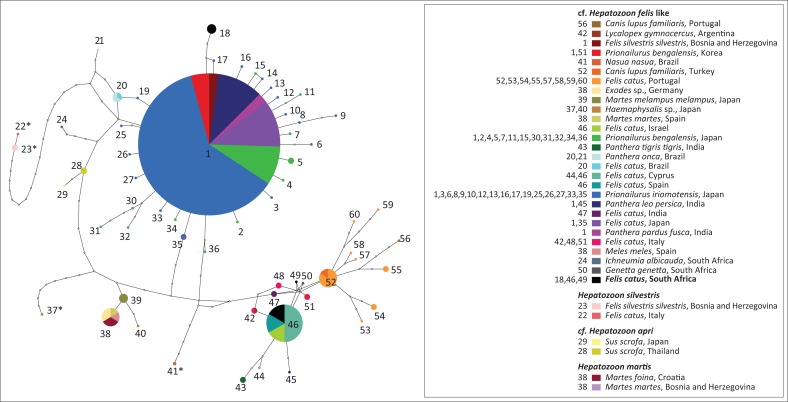
Network showing relationships between haplotypes of *Hepatozoon felis* and other closely related species. Those marked with an aterisk (*) could not be linked to the network with 95% confidence using the TCS methodology. Size of circles represents numbers of haplotypes, and small open circles inferred missing haplotypes. Three haplotypes from South African cats are shown in black and bold.

Of the 16 domestic cat samples examined, 11 gave positive amplifications. Of these, six individuals gave rise to three haplotypes of ‘*H. felis*-like’ sequences that were included in the estimate of phylogenetic relationships and network with other data from GenBank (Figures 1 and 2). The estimates of relationships derived from ML and BI were similar, so a single tree is presented with support value from both analyses (Figure 1). This shows that *H. felis* as currently constituted is not monophyletic. Furthermore, the three haplotypes recovered from cats from South Africa were not closely related. In the network (Figure 2), all ‘*H. felis*-like’ sequences and samples from wild boars cf. *H. apri* could be connected with 95% confidence. *Hepatozoon silvestris* haplotypes could only be included in the network with a higher connection step limit, together with *Hepatozoon* sp. from a coati, *Nasua nasua*, from Brazil, and a tick, *Haemaphysalis longicornis,* collected in Japan (Figure 1). Another domestic cat sample (CatUL115) was positive for *Hepatozoon*, but it was more similar to a distinct lineage typically identified in rodents and snake predators, with a 99% similarity to *Hepatozoon* identified from, among others, jerboa, *Jaculus jaculus* (Maia et al. 2014), or the snake, *Psammophis elegans* (Tomé et al. 2013). This unrelated *Hepatozoon* could not be included in the network with 95% confidence and is not represented in Figures 1 or 2, belonging to a lineage that has been proposed as being potentially a distinct genus (Karadjian et al. 2015). An additional sample (CatR71O14) gave a positive PCR amplification, but the resulting sequence was most similar to another family of haemoprotozoan parasites, Sarcocystidae. The most similar available species on GenBank was *Sarcocystis nesbitti* with 98% similarity. Three samples gave positive PCR amplifications, and the resulting sequences were most similar to parasites of a different apicomplexan order, Piroplasmida, and could be identified as members of the genus *Babesia*. However, the quality of the sequences was poor, and multiple species of *Babesia* are known to infect cats and other felids in South Africa (Bosman et al. 2007). Therefore, these samples could not be analysed further.

## Discussion

Overall, the network and phylogenetic trees revealed extremely high diversity within forms typically assigned to *H. felis*. It seems likely therefore that this represents a species complex. Some tentative biogeographic patterns can be identified. One haplogroup, including haplotype one occurring in several different host species, along with haplotypes that differ from this by just one or two mutations, is mostly found in Japan, India or South America. Most haplotypes from Europe, including Spain, Portugal, Italy, Cyprus but also Israel, form another distinct haplogroup. However, there were exceptions, with, for example, wildcats from Bosnia and Herzegovina having parasites with the common Haplotype 1, while parasites from Indian lions and tigers are more related to the European haplogroup. Haplotypes 29 and 28 were *Hepatozoon* recovered from ticks from wild boars from Japan (Matsuo et al. 2016) and Thailand (Sumrandee et al. 2015), respectively, with the recently described *H. apri* also sharing Haplotype 28 (Hodžić et al. 2018), indicating that all the *Hepatozoon* sequenced from wild boar in this region may correspond to *H. apri*. On the other hand, no *Hepatozoon* were identified from wild boar blood samples collected from Spain (Gimenez et al. 2009).

Among samples from South Africa, haplotype diversity was very high. Their positions are dispersed throughout the network and fall within distinct clades within the phylogenetic trees. Therefore, it seems likely that if *H. felis* as currently constituted represents a species complex, then multiple forms will occur in South Africa. Infection rates also seem to be very high, with 7 out of 16 samples infected with *Hepatozoon*. Although feline hepatozoonosis has been increasingly reported worldwide over recent years, this is still one of the highest proportions reported (Attipa et al. 2017). Despite the small sample size, this warrants further assessment to determine if this high rate is an artefact of sample size or is truly indicative of high infection rates in South Africa. *Hepatozoon* infections are sometimes correlated with other infections, for example, Attipa et al. (2017) identified a significant correlation between infection with *Hepatozoon* and *Leishmania* in cats from Cyprus. Further testing for other feline infectious agents should also be carried out in South Africa to determine if their prevalence is also high. *Hepatozoon canis* infections can show seasonal periodicity (Murata et al. 1993), and this should also be assessed in the *H. felis* complex to determine its impact on reported infection rates. Additionally, identification of the invertebrate final hosts is essential to understand how the parasites spread through the populations.

The finding of another, unrelated type of *Hepatozoon* in domestic cats is unexpected and increases to four known forms recovered from cats (*H. felis, H. silvestris, H. canis*) and an unknown *Hepatozoon* sp. of the ‘Bartozoon’ lineage (Karadjian et al. 2015). Several studies have shown that trophic pathways can lead to infections, with, for example, the pale fox, *Vulpes pallida*, sometimes infected with *Hepatozoon* typical of desert rodents (Maia et al. 2014). The same could be occurring in domestic cats. Whether these are ‘dead-end’ infections, or if the parasites can continue their life cycle in these predator hosts requires further assessment. However, it demonstrates that studies of *Hepatozoon* should preferably sequence positive amplifications, rather than assuming that *Hepatozoon* in felids will correspond to *H. felis* or *H. canis*.

The amplification of a *Sarcocystis* species is not unexpected, as these primers have been shown to amplify these parasites before (Harris et al. 2012) and several species of *Sarcocystis* are known to use cats as definitive hosts (Levine & Tandros 1980). Although additional data are needed to determine to which species this haplotype might belong, the high similarity with *S. nesbitti* warrants further attention as this species can cause illness in humans, but is thought to use snakes as the definitive host and humans as occasional intermediate hosts (Lau et al. 2014). It is therefore particularly important, both from a veterinary aspect and for human health assessments, to investigate how the *Sarcocystis* from a domestic cat may be related to *S. nesbitti*.

To conclude, *H. felis* as currently constituted includes highly distinct genetic lineages, and a detailed assessment with additional and more variable genetic markers is needed to determine how many actual species exist under this name. In the meantime, these lineages can be referred to as the *H. felis*-like complex. Relationships between these *H. silvestris, H. martis* and *H. apri* also need to be elucidated. Domestic cats, as well as being infected by three currently described species of *Hepatozoon, H. felis, H. silvestris* and *H. canis*, can also be infected by an unrelated lineage typically observed in rodents and reptiles that may be another example of infection via a trophic pathway. Therefore, the full list of *Hepatozoon* species that can be found in cats may still be incomplete. Similarly, the finding of an unknown *Sarcocystis* species from a domestic cat indicates that the list of these species infecting cats also needs clarification. Finally, while some general biogeographic patterns are apparent, many areas, including South Africa, can harbour highly distinct genetic lineages.
